# Unlocking Potential: The Potential Impact of ‘Happymakers’ in Alleviating the Labor Shortage in Dementia Care Work: The Paradigm Shift From Care To Well-Being At An Amsterdam-Based Long-Term Care Facility

**DOI:** 10.5334/ijic.8579

**Published:** 2025-07-30

**Authors:** Caroline Van Dullemen, Petra Boersma, Henk Nies

**Affiliations:** 1 Ben Sajet Centre, Amsterdam, The Netherlands; 2 VU University Amsterdam, Departement Public Administration & Political Science, The Netherlands; 3 Faculty of Health, Sports and Social Work, Inholland University of Applied Sciences, Amsterdam, The Netherlands; 4 Endowed professor of Organisation and Policy in Long-term Care (em.), Department of Organization Sciences, VU University Amsterdam, The Netherlands

**Keywords:** integrated care, wellbeing, nursing, dementia, innovation

## Abstract

**Introduction:**

This study aims to identify success factors and challenges of an integrated care model, with an underlying goal of addressing the labor shortage in dementia care. The research investigates the interdisciplinary communication in a long-term care facility in Amsterdam, focusing on the collaboration between the so-called ‘Happymakers’ (non-medically trained staff) and qualified personnel. The Relational Coordination Theory serves as theoretical framework, emphasizing the need for shared goals, knowledge, and mutual respect for effective communication.

**Methods:**

Using qualitative methods, the research involved interviews with thirty staff members in 2022.

**Results:**

Work satisfaction was rated very positively. The collaboration between the ‘Happymakers’ and qualified care workers was generally positive, perspectives on risk perception and task alignment varied. Trust and a culture allowing mistakes were deemed crucial.

**Discussion, Conclusion:**

Overall, the findings suggest that the integration model which includes the paradigm shift from care to well-being, positively influences care quality as well as job satisfaction potentially alleviating the labor market shortage. The study suggests further research on strategies for integrating formal with informal care work and comparative research between integrated dementia care and more traditional, medical oriented types of care.

## Introduction

Currently, almost all European countries face major challenges in providing long-term care (LTC) for older people in terms of access and financial sustainability [1,2,3]. As outlined by WHO, in three-quarters of OECD countries population ageing has outpaced the growth of long-term care (LTC) workers in the past decade [4]. Numerous strategies are depicted for strengthening the resilience of the LTC sector, such as improving working conditions in LTC, training, use of technology, higher wages, better matching skills and jobs, a social dialogue, and legal migration [5,6]. As the European Commission points out in its Care Strategy, there is a need for care workers with soft skills, digital skills and specialized knowledge, for instance in LTC for people with Alzheimer’s disease and other chronic conditions [7].

The context of LTC in the Netherlands fits with this overall picture. The Commission for Working in Healthcare in the Netherlands demonstrated in 2020 that more than 40% of healthcare professionals left the sector within two years after graduation, characterizing it as a ‘leaky sieve.’ Significant shortages of care workers are anticipated [8,9] and therefore the sector is looking for alternative labour market strategies.

Already in 2014 the European Core Competences Framework for working with older people in health and social care has been developed. It describes roles and related competencies for working in LTC. The key roles are expert, communicator, collaborator, organizer, health and welfare advocate, scholar and professional [10].

At the same time, since these last twenty years, a paradigm-shift is ongoing. Person-centred care has been gaining popularity in care, especially with respect to people living with dementia [11,12]. So, whereas LTC and integrated care are supposed to be person-centered and emotion-centered [10], the question is whether these competencies should be concentrated in one category of professionals or whether an interdisciplinary team can also meet the needs of clients with dementia in LTC? A second question is, how the depicted paradigm-shift aligns with labour market opportunities.

### Context of the study

Today, around sixty percent of the people receiving long-term residential care in the Netherlands, are suffering from dementia [13]. Around thirty percent of the persons living with dementia in the Netherlands receive long-term residential care [14]. More specifically, the paradigm-shift towards person centred care, is – amongst others – exemplified in the concept of the so called ‘Social Approach’, which is gaining track in The Netherlands [15]. This approach underscores an integrated, societally embedded, person- and emotion oriented approach to dementia care. This method concentrates on the quality of the relationship between the care workers, the family and friends and the individual persons living with dementia. This necessitates being open-minded and curious, placing greater importance on understanding the person rather than merely focusing on the physical (medical) condition. The focus lies primarily on well-being and giving people with dementia a voice [15].

At a small-scale nursinghome facility in Amsterdam, which was opened in April 2022, the Social approach was put into practice. In the Netherlands, nursing homes deploy multi-disciplinary staff, usually consisting of nursing, personal care, medical, paramedical, psychosocial, and domestic care personnel. However, in this case, a diverse team was brought together consisting of both professional care workers and staff members with alternative (non-care) backgrounds and experiences. This choice was made in order to focus more on wellbeing. Further, the deployment of non-care staff was a necessity because of the shortage of qualified care workers on the labour market.

In the marketing outings the recruiters called for ‘Happymakers’, because of their focus on well-being. These ‘Happymakers’ were selected because of their motivation to work with people with dementia, their individual passion for focussing on wellbeing, communication skills, ability to work in a team, self-reflection, responsibility and empathy. They could have been working for instance like a stewardess, in the hospitality sector, in retail, in marketing or as a pharmacist. As one of the managers stated: “You don’t need a healthcare diploma to give our clients a nice day.”

The current study was carried out in this nursing home facility. The primary focus of this research was to investigate how the Happymakers and the qualified colleagues in this specific form of interdisciplinary collaboration, teamed up during in the first year after the opening of the dementia care facility. The aim of this study is to gain insights into the success factors and challenges of interdisciplinary collaboration between qualified and non-qualified staff. The underlying assumption is that the interdisciplinary collaboration between the Happymakers and their more medically/nursing trained colleagues could serve as a model to deal with the constrained labour market for care workers.

This leads to the following main research question:

How do team members experience their work in terms of job satisfaction, collaborating with colleagues from different professional backgrounds in an integrated team?

In addition a more specific sub-question is posed:

What are the critical success factors in the collaboration between these two groups and in what way the social approach is conducive, what are the perceived challenges, and what are the areas for improvement?

## Theoretical Framework

**Figure 1:**
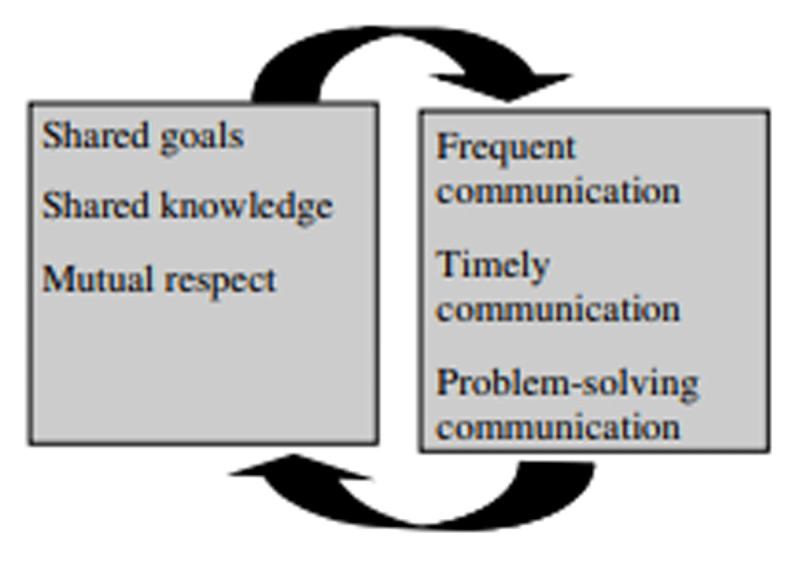
Mutually reinforcing dynamics among the dimensions of relational coordination. *Source:* Gittell (2006).

The study was carried out using the Relational Coordination Theory (RCT) as a theoretical framework. (Hoffer Gittell, 2002, 2006, 2008). RCT focuses on the importance of high-quality communication and strong relationships in achieving effective coordination in the care work processes (Figures 1 and 3). It emphasizes the role of shared goals, shared knowledge, and mutual respect among workers involved in interdependent tasks. The theory suggests that these elements are crucial for ensuring smooth coordination, especially in complex, high-pressure environments like LTC. Relational coordination highlights how these relational dynamics enhance performance, improve outcomes, and boost efficiency by fostering collaboration and problem-solving across different roles and functions.

**Figure 2:**
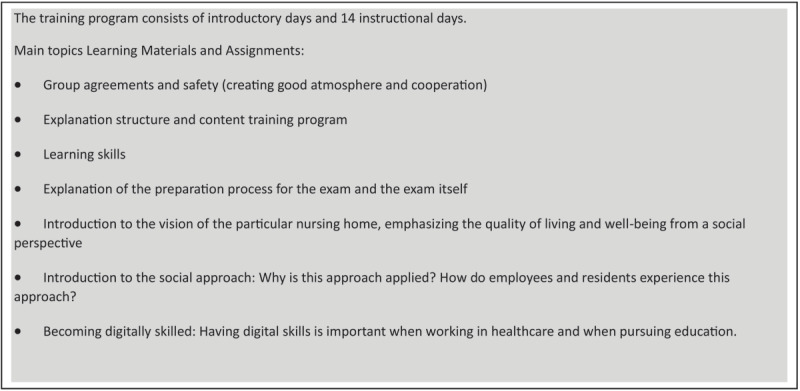
Main topics of 16 wks training program Happymakers as presented by the Regional Training Center.

**Figure 3:**
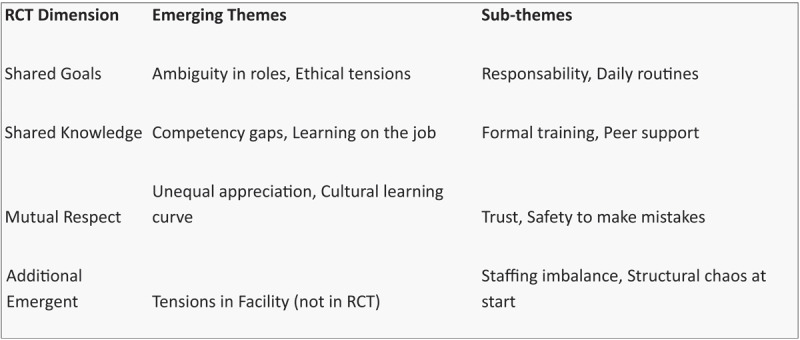
Thematic organisation chart.

The study also focussed on the Integration between the ‘Happymakers’ and the qualified care workers in the context of the Social Approach as a vital element in the paradigm-shift from care to well-being. This approach explicitly aims to break down the boundaries between care and welfare. According to the RCT, interdisciplinary collaboration is reinforced via frequent, timely, problem-solving communication [16,17,18,19,20].

Integrating teamwork and increasing job satisfaction is crucial for enhancing quality of life for people with dementia. In essence, the saying “happy nurses make happy patients” is supported by earlier research [21,22,23,24,25]. Or, in other words, contented caregivers are expected to contribute to a positive work environment, reducing the likelihood of staff turnover, and increasing continuity and stability within the institution [24,25]. Therefore we used the RCT to assess to what extent the care workers are able to effectively integrate their work across the professional boundaries of nursing and social care.

## Methods

### Study design

We conducted a qualitative study using semi-structured interviews between July and December 2022. All participants provided informed consent prior to their involvement in this study, and ethical approval was obtained in accordance with institutional guidelines of the VU University Amsterdam.

We adhere to the participatory adage ‘nothing about us without us’ in our programs and projects. For this research, consistent communication was established with the Regional Older People’s Delegation BetterOld Amsterdam and surroundings.^1^ The members of this delegation played an advisory role throughout the preparation like generating research topics and reviewing the draft report.

### Setting

The study was carried out in a modern care facility designed to accommodate clients with dementia. It houses approximately 36 residents, each with their own private room. This setup ensures personal space and privacy, crucial for their well-being [15]. The facility boasts ample communal areas and a small garden, fostering a homely and supportive atmosphere.

The care team at this facility includes dedicated professionals who provide 24-hour care. Also, professionals, like occupational therapists, physiotherapists, and social workers are employed. A psychologist acts as the lead practitioner overseeing the psychological well-being of residents and coordinating comprehensive care plans with other professionals.

The team comprised of equal shares of Happymakers and ‘Professionals’, i.e. the qualified care workers (50/50). Before they actually started working at the facility, the Happymakers received a secondary vocational training of sixteen weeks (see Figure 2) and later on, the integrated full team received specific training on the concepts and understanding of Social Approach. There were group trainings provided on site as well as online chapters that could be studied on a more individual basis. The guidance in the practice of the specific Social Approach which should steer the process of integrated care and collaboration, was provided by a social consultancy organisation specialized in this approach.

This guidance in Social Approach worked very well in the opening stages of the facility. At the same time, during the launch, the staff faced many challenges. The untimely exit of the initial coordinator and unfilled positions hindered early team collaboration. Only after a new coordinator was appointed did the project gain momentum. The hiring of freelance staff for six months ensured continuity, which was essential for fostering a stable care team and minimizing disruption to residents.

### Characteristics of the participants

In total, thirty interviews were conducted with care workers, which means almost all the staff members. Fifteen Happymakers, ten team members with a qualified background, the coordinator, and four members of the treatment team (the lead clinician (psychologist), geriatrician, physiotherapist, and dietitian).

The team of employees at the LTC facility was very diverse in its composition in terms of professional background, education, work experience, observed age, migration background (see Table 1). Some Happymakers had completed secondary education, while the majority had pursued specified follow-up education, such as graphic design, arts, retail, hospitality, or stewardess training. Note: the exact chronological age was not a key factor in the applied theory, and therefore, it was deemed unnecessary to know the precise age of respondents. Instead, observing age allowed us to distinguish whether we were dealing with a younger or more senior respondent. This helped contextualize their experiences and perspectives, without making assumptions based on exact age, and ensured that the diversity in perceived age contributed meaningfully to the analysis without introducing the risk of unnecessary biases or discrimination.

**Table 1:** Main characteristics of the participants.


CATEGORY	NUM	EDUCATION BACKGROUND	PERCEIVED AGE RANGE	MIGRATION BACKGROUND

Total Participants	30	Diverse backgrounds, including secondary and higher education	Varied	Diverse

Happymakers	15	Mostly secondary education –5 with degrees f.i. economics, biology, art history	Mid-forties to late fifties	Netherlands, Suriname, Eritrea, Marokko

Qualified Team Members	10	Certified healthcare workers (nurses and assistants)	Around thirty to late fifties	Netherlands, Suriname, Curacao, Croatia

Coordinator	1	Health studies	Late twenty	Netherlands

Manager	1	Qualified nurse	Late forties	Netherlands

Treatment Team Members	3	Not specified	Not specified	Netherlands


Additionally, five Happymakers had completed higher education, with academic degrees in fields like economics, biology, or art history. Their observed age range spanned from around mid-forties to the late fifties. In terms of migration background they came from countries like Poland, Eritrea, Surinam. The hobbies and other activities of the Happymakers, such as culinary cooking, (nature) photography, and Shiatsu foot massage, were also important elements of their background. These were often utilized in their work with the residents.

The other part of the team consists of colleagues with a healthcare background, including certified nurses and certified nurse assistants. Their birthplaces varied, with some born in the Netherlands and others in countries like Suriname, Curacao or Croatia. Their observed age range spans from around thirty to the late fifties. Although most of them resided in Amsterdam, some lived in places further away.

### Data collection and analysis

For the semi-structured interviews, a topic list based on the RCT dimensions ‘shared goals’, ‘knowledge’, and ‘mutual respect’ was compiled. Thirty people who had been working from the day the care facility opened its doors, were included in the study: the coordinators, all team members, so the qualified employees, as well as the so called ‘treatment team’ of – amongst – others the specialist geriatric medicine, the psychologist and the physiotherapist. Therefore, no sampling was involved. Collected personal characteristics were education, work experience and place of residence (commuting time). Job satisfaction was operationalized by asking the care workers to rate their current work between 1–10.

In addition to the interviews, ethnographic observations were conducted to gain a deeper understanding of the daily interactions and dynamics within the care facility. These observations involved spending extended periods of time in the facility, documenting interactions, routines, and notable events. Field notes were taken to capture the nuances of these observations. The collected data were analysed according thematic analysis and conducted in several steps: becoming familiar with the data, coding, searching for common themes, reviewing the themes, naming the final themes, selecting appropriate quotes, and reporting.

To enhance reliability, five interviews were independently assessed by two authors and (open and axial). The analysis was assisted by the use of the software program Atlas Ti, version 23. No automated coding, nor AI summaries were used. In the ‘Results’ section, quotations that reflect the responses given by interviewees are presented to illustrate the findings. The quotations are coded based on the respondents’ number and function.

During the coding process, the three RCT theoretical dimensions helped identify and categorize themes within the data, ensuring that the analysis aligned with the theory’s focus on relational dynamics. Themes that emerged, such as effective collaboration or communication challenges, were interpreted through this theoretical lens, ensuring that findings were grounded in the theory’s core concepts.

The following figure illustrates the dimensions, emerging themes, and sub-themes identified during the analysis:

## Results

### Tensions in the facility

In the context of the RCT, the interviews and observations revealed tensions. First, it had to do with the somewhat chaotic launch of the facility. The team coordinator used these words *“I felt like everyone was running around like a chicken at the start. Everyone really wanted to, but there just wasn’t enough the time and everyone was still very searching, right? If you provide structure and frameworks, you will automatically have time for other things and then you also have time to get to know each other well. (…)”*

And secondly, the observed tensions also had to do with an imbalance in terms of numbers between the Happymakers and the qualified care workers, which emerged as a recurring topic of conversation. While the initial assumption was a fifty/fifty distribution, by early 2023, the distribution was thirty percent certified professionals and seventy percent Happymakers. Not all certified employees were positive about this distribution, as expressed by one qualified care worker who stated, *“(…)if they put one certified professional on a unit with three ‘Happymakers,’, I will run away because I won’t take that responsibility.”*

### Job satisfaction

At the same time, this did not seem to influence the level of work fulfilment very much. Job satisfaction is not part of the Relational Coordination Theory, but it seemed interesting to investigate it in the context of the integrated teamwork. Job satisfaction is a contested concept in science because of its inherently subjective nature, varying definitions, and complex measurement challenges. Scholars debate whether it stems primarily from individual factors (like personality) or external conditions (like work environment). Additionally, its impact on organizational outcomes, such as productivity and turnover, remains difficult to quantify consistently [26,37].

Nevertheless, measuring job satisfaction provides an interesting indication of work engagement and overall contentment with the working environment.

The average rate was of 8 out of 10 (range 5–10), indicating a positive attitude to the care work in the LTC facility. Some mentioned that they gave a relatively low mark because of the difficult integration between the Happymakers on the one side, and the qualified care workers on the other side. At the same time they gave a high mark for the way everybody treated the residents with dementia. Overall the rating followed anormal distribution.

One respondent gave a 10 as mark for job satisfaction because *“I am happy with my work. I work with my heart. I am used to help older people. It gives me satisfaction. I always think of my aunt”. (resp. Happymaker)*.

Some of the qualified care workers were a little bit more critical. One of them rated her work with a 7. *“I had hoped that I could have given the work an eight or a nine. Yes, the fact that the residents are no longer in the early stages of dementia plays an important role. We also need to find each other more in the team.” (resp. qualified care worker)*.

Another respondent allocated two ratings: *“I rate the care for older people as 10! But working with the colleagues is still a 5.” (resp. Happymaker)*.

### Shared goals

Having presented the results related to the composition of the teams and the overall job satisfaction, we will now present the data according to the three foundational themes derived from the Relational Coordination Theory: shared goals, knowledge, and mutual respect for effective communication.

In spite of the common context of the Social Approach, there appeared to be ambiguity in *shared goals*. Many employees, qualified care workers as well as Happymakers, saw *taking responsibility and risk signalling* as one of the biggest differences between the Happymakers and the qualified professionals. *“I do think that certified professionals see more risks, and I really appreciate that because it allows us to have a discussion about whether we should take that risk or not” (resp. Happymaker)*.

A point of discussion, for example, is how many residents a staff member could accompany outside. There was also the – in fact – ethical question of whether some residents could go out on their own. Not all team members were enthusiastic about this. In some cases, family members themselves said, *“just let them go, that’s the risk of life”*. Though, particularly colleagues with a healthcare background had serious concerns. Some mentioned that in the past, they had experienced situations where, despite initially indicating that they accept the risks, but in the case of an accident, families still pointed accusatory fingers at the care workers.

Qualified care workers often felt a strong sense of responsibility, not only for the residents but also for the work of the Happymakers. This was linked to their perception of risk. *“We need to have eyes in the back of our heads”*, noted a qualified team member.

Aligned with the tenets of the Relational Coordination Theory, the data indicate a very general division of labour that primarily highlights the limited sharing of goals. *“They have more of the outside world, we have more of the inside world. We have more care plans, medications, orders.” (resp. qualified care worker)*.

Because of the recent start of the facility, there was a lack of established routines and clear task distribution within the team, leading to discussions about structure, agreements, improvisation/flexibility and scheduling. The values and culture of the team yet had to be developed. Although the Social Approach served as the overall framework, it hadn’t been sufficiently incorporated. One respondent vividly described it as, *“I have to think of an orchestra where everyone is just doing their own thing. Essentially, we lack a conductor”*.

The Happymakers supported the Activities of Daily Living (ADL) from a Social Approach perspective. However, there was sometimes friction related to the shared goals. This was particularly the case regarding an important part of ADL, which is how and when to get the residents out of bed. According to the Social Approach, the residents themselves should decide when they want to get up. There is no fixed alarm. However, some qualified care workers considered the waking up of people as an intrinsic part of their role. One of them commented*, “We are considered as ‘old hat’. We pull these people out of bed… They can be left if someone has only physical problems, but these people have dementia. They won’t remember day and night. We provide their structure” (resp. qualified care worker)*.

Additionally, the qualified care staff members were sometimes afraid they would be left behind with the more heavy and dirty work, while the Happymakers do the fun things like taking the residents outside for a walk or a visit at the Zoo.

There was need for a more explicit structure to provide both staff and residents with a sense of stability and safety. Especially the work schedule was a bone of contention. It is made by a few colleagues and was not always conducive for all since there is not much flexibility in it. *“In the care business, (…) you have to do evening and night shifts (…) you cannot say anything but ‘yes’. (…) I think you can block one day, but for the rest you really have to be available. That is quite compelling.” (resp. qualified care worker)*.

However, the majority of all the staff members realized they were in the starting up phase, so most of them expected this working structure would become more explicit after having worked together and integrated their respective activities over a longer stretch of time.

### Shared knowledge

Secondly, there was a debate on shared knowledge, in a number of cases combined with different views on *goals*. Shared knowledge facilitates communication and understanding among team members, while shared goals align their efforts and drive them towards common objectives. Together, they enhance teamwork, efficiency, and the likelihood of success, but sometimes they are difficult to distinguish. Most qualified care workers believed that the Happymakers lacked sufficient experience. It was unclear which of their competencies could compensate for this, or which competencies would bring clear added value to the team, like social skills, culinary, artistic talents or wellness practices. Sometimes, they themselves were also hesitant in this respect. *It’s also about caring and just observing, maybe not from a medical perspective, but just from a feeling “hey, I think something is not quite right here”* a Happymaker mentioned.

*As a photographer, question mark? (interviewer pointed to his alleged talent and experience as an amateur photographer)*.

*“Yes, maybe even as a photographer”* the Happymaker agreed.

Other Happymakers however, wanted to stick to their more social role and they were not particularly interested in the more medical aspects of the work. *“I want to make happy. I do not want to treat wounds or to distribute medication”. (resp. Happymaker)*.

Developing a learning organization requires a safe environment fostered by sufficient social connectedness among the entire staff. Team members of both backgrounds generally felt connected to their colleagues and work. The feeling of appreciation and opportunities for skill development through training seemed to contribute to social connectedness. As time progressed, trust among all team members grew, promoting intrinsic motivation.

*“Yes, I feel appreciated here. At my previous job, that was very different. (…) I feel completely at home here. Sometimes when I’m already at my own home and there is someone short in the facility, I immediately say: Oh, I’ll come. I don’t mind that at all.” (resp. Happymaker)*.

Responsibility and learning were often discussed topics. Most team members agreed on the importance of a learning organization to improve care performance. Some of the Happymakers were interested in the more medical aspects of the work. “*We are learning by doing. I am very interested in wounds myself. So then I ask: ‘what are those black edges, what cream goes on them, etc. And then they also ask: come and have a look, I’m going to take care of the wound again.’ So we learn as colleagues by watching and doing. Yes, mistakes are also discussed. And then we look at what can be done differently.”(resp. Happymaker)*.

The sense of social connectedness and the start of a conducive learning atmosphere could also be linked to the guidance provided by the social consultancy organisation in steering the interdisciplinary collaboration process in the context of the Social Approach. Though the process of supervision hampered somewhat in the beginning, it involved Happymakers and qualified care workers and professionals coming together as an integrated team. The team members contributing distinct competencies and learning the ‘language’ of the Social Approach. The purpose of the training was to develop a common vision, a common language of communication, and thereby, common goals. Many team members, however, experienced the modules as rather abstract. The coordinator had a similar impression and brought forward suggestions for change like shorter modules and more focus on case studies and practical learning.

### Mutual respect

In terms of *shared respect*, there was initially ambiguity among particularly the qualified care workers, because of the perceived added value to the team. Healthcare staff members appreciated Happymakers the most for their different perspective and enthusiasm. Collaboration with the specialized professional staff, amongst others the medical doctor, psychologist, physiotherapist, was going well, with no major differences in appreciation here between qualified care workers and Happymakers. The data also showed confidence in an improving future collaboration between Happymakers and qualified care staff. *“Yes, I enjoy the Happymakers. Yes, that is really not an exaggeration. Why? (…) I have a lot of confidence in them and if they are serious and open they can learn things well vary rapidly.” (resp. qualified care worker)*.

The Happymakers had a close bond amongst themselves due to the fact they had made a serious career change, they often had experienced care issues in their personal lives and they followed a joint training. *“We also keep in touch outside of work. We recently went to the Zoo. We complement each other well, I do that, you do this. It’s very easy, of course. It was that way from the beginning.” (resp. Happymaker)*.

Due to their close bond, they shared more common values, which sometimes left the qualified care staff feeling somewhat excluded. Additionally, some staff felt that the “Social Approach” implied their work was outdated or inferior because they followed traditional care methods. One staff member remarked, *“(…) the Happymakers were all fantastic, and we were just the old folks….” (resp. qualified care worker)*.

In sum, the dynamics in the integrated team were not always mutually reinforcing. Frictions and discussions took place about structure, agreements, and flexibility. Nevertheless, Happymakers were generally appreciated for their alternative viewpoint, their energy and devotion. But they were also seen as inexperienced and lacking in identifying residents’ care needs. Finding the right balance between the care and well-being perspectives was an ongoing challenge, with an underlying debate about competencies that were needed. Echoing the principles of the Relational Coordination Theory, the team exhibited varying perspectives on allowing and discussing “mistakes” as a means to stimulate mutual respect, operational improvements and cultivate a culture of trust. Within these dynamics, the absence of *shared goals* became apparent, only partially related to differences in shared knowledge.

A few team members had the feeling that there were certain colleagues who do not tolerate any mistakes. *“No, you cannot make any mistakes. That is interpretated so badly by people who feel so superior (…) I think it’s a shame that you give people the feeling that they can’t make any mistake, because first of all, you scare people with it. Secondly, I have also heard that ’hey, I’m not going to tell anyone about a mistake anymore, because then everyone will know about it’. No, that could be a bit better, among our team” (resp. qualified care worker)*.

Other team members (qualified and non-qualified) referred to *shared respect*. They – in contrast – had the feeling that there is a more open culture in which errors or slipups could be shared. *“Yes there is certainly enough trust. We just talk about these things”. (resp. Happymaker)*. Some Happymakers or qualified care workers had standpoints in between. They might formally report an Medical Incident with a Client (MIC), but they did not feel surrounded by a genuine safe environment.

*“Making mistakes? I don’t know. MIC reports on medicines? There is no safe feeling at all!” (resp. qualified care worker)*.

The majority of team members were highly enthusiastic about the way the former coordinator steered the team with openness and support. Her frequent presence on the work floor was appreciated, enhancing her accessibility and fostering a culture where team members felt comfortable addressing issues, ranging from minor mistakes to more significant errors. The coordinator not only effectively structured work processes but also served as a safe haven for staff members. This mutual openness was evident in an incident where an individual proactively shared a mistake, creating an environment where such transparency was encouraged and positively reinforced.

Despite the ongoing discussions and examinations surrounding the exact nature of the relationship between these two distinct groups—Happymakers and qualified care workers—it was clear that there was a prevailing optimism about their prospects for working together harmoniously in the future. Almost all of the staff members mentioned the potential for enhanced clarity in communication and anticipated improved collaboration as the team’s mutual understanding and acquaintance deepened over time.

## Discussion

The objective of this study was to acquire a deeper understanding of the factors contributing to success in interdisciplinary cooperation in LTC facilities for people with dementia and which obstacles were encountered. The Happymakers can be viewed as a specific form of lateral entrants to the care domain. The fundamental premise was that the interdisciplinary collaboration between the Happymakers and their colleagues with a professional medical background could offer a blueprint for recruiting unlocked potential in the labour market of dementia care workers.

The most prominent result of this study was the high level of job satisfaction – in the interviews the staff members rated the work on average an eight on a scale between one and ten. Other results implied shared goals instigated by training, and mutual respect in the context of diversity as critical success factors. The challenges stemmed from issues of imbalance, insufficient shared knowledge, structural ambiguity, role perceptions, and communication dynamics, as well as the phase of team-development. As Miller and De Andrade (2021) point out, different professions have different values and ethical codes, which need to be addressed in order to implement a value based practice. By addressing these challenges and aligning the various professional cultures into one team the coordinator was on her way to building a more effective and harmonious collaborative environment. At the same time, a shared view on the role of the coordinator could be supportive to a tighter team integration. The Social Approach did serve as a framework for interdisciplinary collaboration, as well as for the intended paradigm-shift. However, it had been implemented in a relatively late stage and a rather fragmented way in this particular setting. In that sense, it did not effectively align all competences of the varied staff from scratch on.

Earlier studies showed that management support and person-centred-care practices positively correlate with improved staff satisfaction, staff retention and quality of care. A review by Rajamohan et al. [27] suggested that job satisfaction in LTC facilities is positively related to consistency in the quality of care delivery and increased quality of life among residents in nursing homes. The review showed that person-centred-care interventions (which encompasses a large part of the ‘Social Approach’ as well as the widely adopted principles for person-centered care for people with dementia as formulated by Kitwood [11] and training representing the key concept of workplace resources have a positive impact on nursing home staff job stress and satisfaction [27].

In the present facility, the organizational culture was seen as somewhat too flexible and lacking clear rules and procedures. However, it was also recognized as evolving towards a more cohesive structure with shared goals. The results of this study showed the team members were, after an initial leadership crisis, very satisfied with the current leadership. As frequently mentioned in the literature, leadership is key for successfully integrating multi-disciplinary learning, culture change, relational work and team-work with a variety of professionals [28,29]. Also, the importance of ethical principles appeared to be underlying many disputes that had to be resolved in the team. Joint values and ethical principles are not only important for integrating interdisciplinary teams, but also for job-retention for regular LTC staff.

In fact, a process of multidisciplinary working evolved into a direction of what the researchers point out as transdisciplinary learning [30]. It remains to be seen whether siloed learning of nursing related expertise should be part of the competence profile of all care workers in LTC, following the European Core Competences Framework for working with older people in health and social care [10]. The findings of this study suggest that staff with different vocational backgrounds can also possess or acquire a significant range of competences needed. This implies new labour market opportunities for both LTC ánd integrated care.

Furthermore, the positive attitude of staff toward future collaboration in the facility that was studied, aligns with the principles of Relational Coordination Theory which emphasize the significance of shared goals, shared knowledge, and mutual respect in promoting effective communication and integration among team members. As such, although the shared goals and knowledge were not yet properly entrenched. The optimism within this hybrid team formation is not only indicative of a potential solution to the tight labour market but also an embodiment of the Relational Coordination Theory’s core principles in action. Besides this, diversity in goals, and underlying values and knowledge were observed. One can argue, that in interdisciplinary teams, this should not be a problem, but an asset. If only similar knowledge would be shared, there would be no reason for an interdisciplinary composition of the team. The various disciplines may not only have a different knowledge base, but also partly different goals with different underlying values. This may be a barrier, or an enrichment, provided there is mutual respect. As the team developed, this gradually became apparent in the course of time. It may have contributed positively to job satisfaction.

In short, despite the initial challenges, the positive undercurrent of the shown optimism regarding future collaboration within the hybrid team formation suggests that it has the potential to address the challenges posed by the current labour market for care work. This promising outlook not only enhances the work environment but also aligns with the principles of effective collaboration as advocated by the Relational Coordination Theory.

### Contribution to theory validation

The study results contribute to the Relational Coordination Theory by providing empirical evidence and real-world examples that align with the core principles of the theory. Specifically, the findings support and reinforce several key aspects of the theory:

Shared Goals and Mutual Respect: The study’s observations of increasing cohesion and trust in collaboration between Happymakers and qualified care staff resonate with the theory’s emphasis on shared goals and mutual respect as drivers of effective collaboration. The challenges faced in balancing care and well-being perspectives highlight the importance of understanding and managing interdependencies between different roles, aligning with the theory’s focus on these elements.

Shared Values: The study’s identification of issues related to task distribution and the need for a more explicit structure for stability and safety corresponds with the theory’s emphasis on the importance of clear role definitions and task integration among team members. These findings underscore the shared values of clarity, structure, and safety within the team.

Shared Knowledge: The study’s findings regarding differing perspectives on handling (-medical- knowledge) mistakes, learning opportunities and variations in feedback and error communication are consistent with the theory’s recognition of the role of effective communication and problem-solving in achieving relational integration.

In conclusion, the study’s results support and enrich the Relational Coordination Theory by providing practical insights and more operational examples that demonstrate how the theory’s principles can be applied in the context of innovative dementia care as well as interdisciplinary working. This empirical validation reinforces the theory’s relevance and applicability in promoting collaboration, staff engagement, and resident satisfaction, ultimately benefiting healthcare organizations if other similar research also points into this direction.

### Strengths and limitations

All Happymakers and qualified care workers who have been in charge since the opening of the facility were interviewed, providing a comprehensive picture of the experiences in terms of participation from all involved parties. Reliability was enhanced by having all respondents interviewed by the same researcher using the same protocol, and observations added to the validation of the data, fostering a safer interview atmosphere.

Data analysis was guided by the Relational Coordination Theory, which provided a framework for structuring the findings around its core principles of shared goals, shared knowledge, and mutual respect. The study contributes to this theory by demonstrating how the Happymakers model fosters these principles within the context of a newly established LTC facility. For instance, the high job satisfaction among staff suggests strong alignment with shared goals, as interviewees repeatedly highlighted their collective commitment to improving resident care. This was reflected in statements such as “*We all knew we were building something special together*,” illustrating a shared understanding of their roles.

To further promote reliability, five interviews were analysed by two independent researchers, with minimal disagreement between their interpretations. However, this study’s limitations include its restricted sample, as it focused on one ward of the nursing home, excluding residents with dementia or their family members from interviews. Conducting the majority of conversations on-site might have influenced responses due to social desirability bias. Nonetheless, the researcher spent substantial time observing and participating in activities within the care facility, helping to mitigate this limitation. However, these ethnographical observations could have been more extensive to contextualize the outcomes of the interviews.

Another limitation is the timing of the study, which took place during the early months of the facility’s opening. The high job satisfaction observed might be partly due to the enthusiasm of working in a new organization, where staff is typically highly motivated. This is supported by literature highlighting the correlation between motivation and job satisfaction in new roles or facilities [31,32,33,34,35]. It is important to note this early phase, as the initial high satisfaction may decrease over time due to staff turnover and evolving team dynamics.

### Suggestions for further research

During the data analysis phase, several broader research questions surfaced, providing avenues for future exploration:

1. *Translating further care integration with informal care*. To what extent can the integrative model observed between qualified care workers and the Happymakers in institutional care settings be applied to further collaboration with informal care like family members, friends and volunteers?
2. *The paradigmshift from care to well-being exhibits promising potential for alleviating pressure on the labour market; however, it necessitates subsequent inquiry, particularly in the way these integrated teams could serve as a vehicle to person centred care in already existing long term care facilities*.

These broader research questions encompass a range of critical topics within healthcare and workforce dynamics, offering opportunities for in-depth investigations and contributing to the advancement of knowledge in these domains. These topics can be associated with Carlile’s [36] research on boundaries in organizational theory, examining the management of knowledge across boundaries in environments where innovation is desired. Depending on the level of innovation and ambiguity in the organization’s purpose and environment, not only (medical) knowledge transfer but also translation and even transformation of values may be required.

## Conclusions

The main research question of the study was how team members experienced their work in terms of job satisfaction, collaborating with colleagues from different professional backgrounds in an integrated team caring for people with dementia living in a nursing home. The team consisted of care workers with diverse backgrounds and educational levels, care and non-care. This study indicates that interdisciplinary team members currently give their job satisfaction a high grade, leading to engagement and job satisfaction among staff, conceivably positively impacting the quality of care and therefore resident well-being. The finding indicates that collaboration between staff with specific medical/nursing professional training and those recruited laterally, with different professional backgrounds and only short-term training, contributes to the aspired paradigm shift from care to well-being. The results suggest furthermore that this approach could potentially contribute to a solution to the tight labour market for dementia care workers.

## Footnotes

The ROD Amsterdam e.o. is one of eight regional networks for older people that join forces in the Netherlands to promote inclusive participation of older people. This collaboration ensures that older people are seen and heard from all over the country. https://bensajetcentrum.nl/regionale-ouderen-delegatie/.
